# Effects of Phenols from Olive Vegetation Water on Mutagenicity and Genotoxicity of Stored-Cooked Beef Patties

**DOI:** 10.3390/antiox13060695

**Published:** 2024-06-06

**Authors:** Dario Mercatante, Sarah Curró, Patrizia Rosignoli, Vladimiro Cardenia, Beatrice Sordini, Agnese Taticchi, Maria Teresa Rodriguez-Estrada, Roberto Fabiani

**Affiliations:** 1 Department of Agricultural and Food Sciences, Alma Mater Studiorum—University of Bologna, 40127 Bologna, Italy; dario.mercatante2@unibo.it (D.M.); maria.rodriguez@unibo.it (M.T.R.-E.); 2 Department of Comparative Biomedicine and Food Science, University of Padova, 35020 Legnaro, Italy; sarah.curro@unipd.it; 3 Department of Chemistry, Biology and Biotechnology, University of Perugia, 06129 Perugia, Italy; patrizia.rosignoli@unipg.it (P.R.); roberto.fabiani@unipg.it (R.F.); 4 Department of Agricultural, Forest and Food Sciences, University of Turin, 10124 Torino, Italy; vladimiro.cardenia@unito.it; 5 Department of Agricultural, Food and Environmental Sciences, University of Perugia, 06126 Perugia, Italy; beatrice.sordini@unipg.it; 6 Interdepartmental Centre for Industrial Agrofood Research, Alma Mater Studiorum—University of Bologna, 47521 Cesena, Italy

**Keywords:** cooked beef patties, olive by-products valorization, phenolic extract, mutagenicity, genotoxicity, oxysterols, heterocyclic amines

## Abstract

This explorative study aimed to assess the mutagenicity and genotoxicity of stored-cooked beef patties formulated with and without phenols (7.00 mg of phenols/80-g patty) extracted from olive vegetation water (OVW), as related to the formation of cholesterol oxidation products (COPs) and heterocyclic amines (HCAs). The patties were packaged in a modified atmosphere, sampled during cold storage (4 °C) for 9 days, and grilled at 200 °C. The genotoxicity was evaluated by the Comet assay. The patty extract was found to be genotoxic on primary peripheral blood mononuclear cells (PBMCs), while no mutagenicity was detected. The addition of OVW phenols significantly decreased the genotoxicity of the patty extract and reduced the total COPs content in stored-cooked patties (4.59 times lower than control); however, it did not affect the content of total HCAs (31.51–36.31 ng/patty) and the revertants’ number. Therefore, these results demonstrate that the OVW phenols were able to counteract the formation of genotoxic compounds in stored-cooked beef patties.

## 1. Introduction

Diet is widely recognized as a major determinant in the onset and progression of some chronic degenerative diseases, like cardiovascular diseases, cancer, and diabetes [1]. Regarding cancer, it has been estimated that a striking percentage of cases are attributed to dietary factors (10–20%) and could be prevented by changing dietary habits [2]. However, different foods can act as both risk and preventive factors for carcinogenesis. Among the risk factors, the International Agency for Research on Cancer (IARC) has allocated red meat consumption as a probable human carcinogen (Group 2A) and processed/cured meat as a carcinogen (Group 1) [3,4,5]. These unhealthy properties have been linked mainly to the addition of nitrates/nitrites and the associated formation of *N*-nitroso compounds (NOCs), as well as to the generation of lipid oxidation compounds (i.e., malondialdehyde and 4-hydroxy-nonenal from the degradation of unsaturated fatty acids) and cholesterol oxidation products (COPs). COPs also play a pivotal role in the development of atherosclerosis and various chronic diseases [6,7], like Alzheimer’s disease [8], Parkinson’s disease [9], and neuroinflammation [10]; lately, they have also been implicated in human inflammatory bowel diseases [11]. Cholesterol can be oxidized by enzymatic and non-enzymatic pathways (autoxidation and photosensitized oxidation), which are highly dependent on processing and environmental conditions (oxygen concentration, light, temperature/time). Indeed, sterol oxidation is a multi-factorial process that is affected by food quality, manufacturing procedures, storage conditions, and cooking methods [12]. In particular, high-temperature cooking methods (such as grilling and barbecuing) have been shown to result in the accumulation of COPs in red meat steaks and patties [13]. Broncano et al. [14] grilled pork steaks at 190 °C for 4 min and found that cooking doubled the COPs’ level in the grilled samples (337.6 μg/100 g of meat) compared to the raw ones. Besides NOCs and COPs, there are other carcinogenic chemical compounds that are also produced during meat processing and cooking, such as polycyclic aromatic hydrocarbons (PAHs; classified into different IARC groups (1, 2A and 2B) depending on the type of PAH) [15] and heterocyclic aromatic amines (HCAs) [16]. The latter are known to be mutagenic and possibly carcinogenic (groups 2A and 2B of the IARC classification), and the amount produced depends on the amount of protein present, the cooking method, and conditions (temperature and time). High-temperature cooking methods produce the highest levels of HCAs [17]. The most common HCAs (2-amino-1-methyl-6-phenylimidazo[4,5-*b*]pyridine (PhIP), 2-amino-3-methylimidazo[4,5-*ƒ*]quinoline (IQ), 2-amino-3methylimidazo[4,5-*ƒ*]quinoxaline (MeIQx), and 2-amino-3,4,8-trimethyl-imidazo[4,5-*ƒ*]quinoxaline (4,8-DiMeIQx)) are produced by the amino acid’s pyrolysis or by a complex reaction that involves the product of the thermal decomposition of amino acids (pyridine or pyrazine) and creatinine [18]. PhIP is the most common dietary HCA found in the human diet, but IQ, MeIQx, and DiMeIQx are some of the most powerful mutagenic substances analyzed with the Ames/Salmonella assay [19]. Numerous epidemiology investigations have suggested that a high consumption of well-done meat may raise the risk of cancer in humans, mainly ascribable to the presence of high quantities of HCAs [20]. To limit the impact of dietary COPs and HCA on cancer risk, it has been advised to formulate food products with bioactive compounds having a free radical scavenging activity, such as phenols [18]. This class of compounds are widely present in virgin olive oils; in particular, for the aglycon forms of oleuropein and of demethyloleuropein and their derivatives (hydroxytyrosol (3,4-DHPEA) and tyrosol (*p*-HPEA)), in vivo anticancer activity [21,22] has been demonstrated, together with the in vitro ability to defend against the DNA damage induced in different cellular systems by diverse compounds [23]. Furthermore, as recognized by the EU Regulation No. 432/2012 [24] for the authorized health claim, the dietary intake of virgin olive oil phenolic compounds significantly contributes to reducing the levels of oxidized low-density lipoproteins (LDL) in human plasma. However, most of the phenolic compounds present in olives are not transferred to the oil but remain in the olive pomace and the olive vegetation water (OVW), which are the solid and the liquid by-products, respectively, of the mechanical extraction of olives [25,26]. For this reason, olive phenols are often recovered from the by-products of the olive oil chain and used as extracts; in fact, they have already been tested in different products of animal origin, demonstrating their efficacy as antioxidants [27,28,29,30,31,32,33].

Therefore, the purpose of this work was to assess the ability of an OVW phenolic extract to counteract the formation of COPs and HCAs in grilled beef patties subjected to a shelf-life study, as well as to evaluate its impact on the mutagenicity and cytotoxicity of such cooked meat products.

## 2. Materials and Methods

### 2.1. Phenolic Extract (PE)

PE was produced from fresh OVWs as spray-dried powder, as reported by Mercatante et al. [34]. The content of total phenols of the PE was 25.7 mg/g of dried product (determined as reported in paragraph 2.3), with 3,4-DHPEA-EDA (oleacein), 3,4-DHPEA, verbascoside, and *p*-HPEA being the main phenols, as reported by Miraglia et al. [35].

### 2.2. Preparation of the Beef Patties

Two different beef meat cuts (shoulder and rump muscle trimming) from Spanish adult bovines, were used for the preparation of the beef patties. After being trimmed and minced (6-mm diameter), the meat was combined with salt (0.64 g/patty) and starter cultures (Bactoferm^®^ and SafePro^®^; Chr. Hansen GmbH, Hørsholm, Denmark). The resulting meat batter was then split into two parts: (1) Control, meat batter added with maltodextrin (0.28 g/patty; maltodextrin glucidex 19, Roquette, Beinheim, France); and (2) P1, meat batter added with PE (≈7.00 mg phenols/patty). Beef patties of about 80 g each were then molded. Two by two patties were placed in trays, covered with a three-polymer packaging film with anti-UV and anti-fog properties (62-μm total thickness; Guillin 5025N, Usmate-Velate, Italy), and saturated with a modified atmosphere mixture (50% nitrogen, 30% carbon dioxide and 20% oxygen). To imitate retail storage conditions, the beef patties’ trays were placed in a bench fridge (4 °C) for 9 days and exposed to 12-h fluorescent illumination. The beef patties were sampled immediately after production, 6 days, and 9 days of storage (T0, T6, and T9, respectively), and grilled with an electrical grilling plate at 200 °C (4 min/side) to attain 70 °C at the core. The beef patties were chilled (20 °C/5 min) before being placed in a blast chiller (−40 °C/15 min), vacuum-sealed in a plastic bag, and stored at −80 °C. Two batches of beef patties were independently prepared.

### 2.3. Phenols Analysis

The extraction of the phenolic compounds from the PE was carried out by solubilizing 20 mg of the sample in 10 mL of water, which were then filtered through a 0.2-µm PVDF syringe filter (Carlo Erba, Milan, Italy) and analyzed by HPLC-DAD according to Selvaggini et al. [36]. A calibration curve with pure analytical standards was built for each compound here evaluated. The results are expressed in mg/g of PE. Each measurement was carried out in duplicate.

The extraction of the phenolic fraction from the beef patties was performed by solid-phase extraction (SPE) as suggested by Miraglia et al. [35]. The HPLC-DAD analysis of the purified phenolic extract was carried out as suggested by Selvaggini et al. [36]. Total phenols were calculated as the sum of the detected and quantified single phenolic compounds (3,4-DHPEA-EDA, 3,4-DHPEA, *p*-HPEA and verbascoside). Each measurement was performed in duplicate.

### 2.4. Lipid Extraction

A modified version of the Folch method [37] was used to extract the lipid fraction from 5 g of beef patties. Three independent replicates for each sample were carried out.

### 2.5. Determination of Cholesterol Oxidation Products (COPs)

COPs from the beef patties were extracted by cold saponification of the lipid extract, purified by SPE-NH_2_, and injected as trimethylsilyl derivatives in Fast GC/MS as reported by Barbieri et al. [28]. The mass spectra and retention times of COPs commercial standards (Avanti Polar Lipids (Alabaster, AL, USA); Sigma Chemical (St. Louis, MO, USA); Steraloids (Newport, RI, USA)) were used for the identification and quantification of the GC-MS peaks. The COPs were quantified in the single ion monitoring (SIM) mode, by using calibration curves built for individual oxysterols using the internal standard method (19-hydroxycholesterol, Steraloids). The amounts of the single COP were expressed as μg/patty, considering a patty weight of 80 g. Three independent replicates for each sample were carried out.

### 2.6. Determination of HCAs

The extraction of HCAs from the beef patties was carried out according to Turesky et al. [38]. The analysis of the aromatic amines was performed by UHPLC-DAD-Q-TOF/MS; the instrument was an UHPLC system mod. 1260 Infinity (Agilent Technologies, Santa Clara, CA, USA), composed of a binary pump, a degasser, an autosampler, and a thermostated oven coupled to a quadrupole-time-of-flight (Q-TOF) mass spectrometer with Electrospray ionization (Dual ESI), model Agilent 6530 Accurate-Mass Q-TOF LC/MS (Agilent Technologies, Santa Clara, CA, USA). The HPLC column was a Zorbax Eclipse Plus-C18 (100 mm × 2.1 mm, 1.8 μm, Agilent Technologies). Five μL of the sample were injected, and a mixture of H_2_O:CH₂O₂ (99.9:0.1, *v*/*v*) (solvent A) and CH₃CN:H_2_O:CH₂O₂ (90:9.9:0.1, by volume) (solvent B) at a flow rate of 0.4 mL/min was used for eluting the HCAs. The elution gradient was: 0 min, 98% phase A and 2% phase B; in 8 min, 40% A and 60% B; in 2 min, 0% A and 100% B maintained for 20 min. The mobile phase turned back to the starting conditions and was maintained for 7 min to allow equilibration. The total HPC run lasted 37 min of which 30 min was acquisition time.

A *m*/*z* range of 100–1600 was used for mass spectra acquisition in the positive-mode ESI ionization, at a scan rate of 1.0 spectra/s. The Dual ESI source parameters were: gas temperature, 350 °C; drying gas flow, 9 L/min; nebulizer pressure, 40 psig; capillary voltage, 3500 V; fragmentor, 120 V; skimmer, 65 V; 1 RF octapole, 750 V. Data were collected in all ion MS/MS mode by using full scan and in MS/MS with collision energies ranging from 30 V to 35 V. The acquisition in MS/MS was performed to confirm the real presence of the studied molecules in order to avoid false positives; for this reason, qualifiers corresponding to the masses of other ions produced by the MS/MS fragmentation of the analyzed compounds were used. HCAs were quantified using calibration curves for each compound by means of the internal standard method; the results were expressed as ng/g of sample. Two independent replicates for each sample were carried out.

### 2.7. Isolation of Mononuclear Cells from Peripheral Blood

Mononuclear cells (PBMCs) were isolated from leucocyte-enriched human peripheral blood using Histopaque 1077, according to Rosignoli et al. [39]. The layer at the interface with Histopaque-containing PBMCs was recovered and washed twice with simple RPMI 1640 medium. The recovered cells were counted with the trypan-blue exclusion method and density adjusted to 1 × 10^6^ cells/mL to use in the different tests.

### 2.8. Treatment of PBMCs with Meat Extracts and Cytotoxicity Analysis

The PBMCs were treated with the meat extracts in complete RPMI 1640 medium (10% FCS, 2.0 mM L-glutamine, 100 U/mL penicillin and 100 U/mL streptomycin). The incubation was carried out under 5% CO_2_ at 37 °C for 30 min. The cells were then recovered to evaluate the viability by the trypan-blue exclusion method and the DNA damage by the comet assay. The cytotoxicity tests were carried out with a Burker chamber as previously reported [39]. None of the treatments induced any cytotoxic effect; indeed, the viability detected was always above 85%.

### 2.9. Single-Cell Gel Electrophoresis (SCGE or Comet Assay)

The SCGE assay was performed using 50–100 µL aliquots of the cell suspension as previously described [39]. After the inclusion of the cells in agarose and lysis, the slides were treated with freshly made buffer (1 mM Na_2_EDTA, 300 mM NaOH) and incubated for 20 min for DNA unwinding. After electrophoresis (20 min at a fixed voltage of 25 V), the slides were washed with neutralization buffer (0.4 M Tris–HCl, pH 7.5) and stained with ethidium bromide (20 µg/mL). Before analysis, the slides were maintained at 4 °C in the dark. All the procedure was performed under a red light to avoid further DNA damage.

### 2.10. Comet Detection

Twenty-four hours after being stained, the cells were examined under a fluorescent microscope (Zeiss, Jena, Germany) at 400× magnification as reported by Collins [40]. The morphology of the comets was determined by a PC image-analysis (Comet assay II, Perceptive Instruments, Bury Saint Edmund, UK). Many parameters are provided by the software, including the “Tail Intensity%”, which is the percentage of fluorescence intensity in the tail compared to the overall comet intensity [41].

### 2.11. Ames Test

The Salmonella typhimurium his reversion test (Ames test) was performed according to previous procedures [42].

### 2.12. Statistical Analysis

The data were processed with the XLSTAT software version 7.5.2 (Addinsoft, Paris, France). The chemical data are presented as the mean values of three independent replicates of each analysis. Firstly, to test the data’s normal distribution (*p* < 0.05), the Shapiro-Wilk method was utilized. Thereafter, a two-way analysis of variance (ANOVA) was used to analyze the data, setting formulation (F), storage time (S), and their interaction (F × S) as factors. A post hoc Tukey’s honest significance test at a 95% confidence level (*p* < 0.05) was used to distinguish the means of statistically diverse parameters. To compare C and P1, the student’s *t*-test (*p* < 0.05) was used at each time point. To explore possible correlations among all the tested parameters, principal component analysis (PCA) with a Varimax rotation was performed.

## 3. Results and Discussion

The role of upcycling in processed meat and its impact on consumer acceptance is a relevant aspect in the food industry. Upcycling involves transforming food waste and by-products into new added-value products, which can help in mitigating the food waste issue and contribute to a more sustainable food chain. Several studies have examined the factors that could influence consumer acceptance of upcycled processed meat products [43]. Key findings include neophobia, awareness of food waste, environmentalism, age, gender, education level, and knowledge of upcycled foods [43]. Another aspect that can also significantly impact consumer acceptance is the higher level of processing required for upcycled foods, as consumers generally prefer less processed products [44]. Last but not least, consumers are also concerned about the safety of the upcycled products, since they derive from food waste or by-products [44], so monitoring the safety of food products formulated with natural extracts from agricultural by-products is a relevant issue.

### 3.1. Impact on Total Phenols in Stored-Cooked Beef Patties

Table 1 shows that the total phenols in stored-cooked P1 significantly decreased by 3.5 times from T0 to T9. The combined effect of storage time and cooking on phenols’ degradation is clearly related to their oxidative susceptibility [45], as extensively reported by previous studies in different storage and cooking conditions [27,28,31,35].

### 3.2. Formation of COPs in Stored-Cooked Beef Patties

The total COPs levels in stored-cooked beef patties varied from 229.72 µg/patty to 1359.33 µg/patty (Table 1), being 4.59 times higher in C than in P1, after 9 days of storage. In general, the total COPs significantly increased in all the cooked samples with increasing storage time, but the addition of phenols was able to effectively limit cholesterol oxidation. In fact, olive phenols have been proven to have good free radical scavengers, and thus they have been lately used (as extracts from olive by-products) to stabilize meat products [27,28]. Moreover, *orto*-diphenols from olive oil can also chelate metal ions, thus reducing the Fenton reaction and its impact on the oxidation process [46]. Considering individual COPs, the most abundant were 7β-hydroxycholesterol (7β-HC) and 7-ketocholesterol (7-KC) (76.29–428.64 and 76.15–547.71 µg/patty, respectively), followed by 5β,6β-epoxycholesterol (5β,6β-EC) and 5α,6α-epoxycholesterol (5α,6α-EC) (51.55–283.72 and 17.00–77.98 µg/patty, respectively); this COPs profile is similar to that reported by Barriuso et al. [47].

The main COPs detected in our study were those generated from 7-hydroperoxides through a monomolecular reaction pathway, while those produced through a bimolecular reaction between a hydroperoxyl radical and cholesterol were present at lower concentrations, especially CT since it requires water for its formation. In fact, depending on the cooking conditions (time/temperature), grilling is known to dehydrate meat to a large extent during cooking [47]. Most of the COPs were significantly influenced by product formulation, storage time, and their interaction, except for 5α,6α-EC and CT, which were only affected by the product formulation.

In general, cholesterol in beef meat is highly susceptible to oxidation, especially when meat is stored under commercial retail conditions and subsequently subjected to high-temperature cooking in an oxygen-rich environment [47]. Oxysterols tend to form and accumulate in stored-cooked meat, but their profile and amounts are highly dependent on the storage (packaging, irradiation) and cooking conditions used [47]. During autoxidation and thermally induced oxidation, hydroperoxides in C-7 are mainly formed, which can undergo a dismutation and lead to the formation of 7-hydroxyl and 7-keto derivatives. In particular, 7-KC is often the most abundant COP and has therefore been designated as a reliable indicator of cholesterol oxidation in non-cooked food (about 30% of the total COPs content) [48]; as confirmed by our data, 7-KC does not accumulate in extensively cooked meat (200 °C/more than 5 min/side) (Table 1): in fact, 7-KC may break down, dehydrate, and/or react with amino-containing molecules to form Schiff bases [48]. Dietary cholesterol oxides, especially 7-KC and 7-HCs, have been shown to have significant adverse effects on the human metabolism and, at relatively high concentrations, to have significant cytotoxic properties; COPs have been implicated in cancer, cardiovascular diseases, neurodegenerative disorders, inflammatory bowel diseases, and retinopathies [9]. Although there are no toxicity limits for COPs, the threshold of toxicological concern (TTC) for unclassified substances that are potential DNA-reactive mutagens and/or carcinogens (0.15 µg per person per day) [9], is currently used as a reference upper limit for COPs’ consumption in an attempt to reduce their intake [9]. Considering our results, all samples of stored-cooked beef patties were above this limit as single compounds and as the sum of the 5 COPs detected here (>0.75 µg per person per day). On the other hand, neither the EU nor the US currently have an accurate, detailed knowledge of the dietary exposure and consumption of COPs, so it is still unknown whether the current dietary intake level in the population is safe; for this reason, it is important to limit the intake of these substances and reduce their presence in foods [49].

### 3.3. Formation of HCAs in Stored-Cooked Beef Patties

Protein-rich foods cooked at high temperatures produce HCAs, which are potent mutagenic and carcinogenic compounds [19]. To limit and inhibit the formation of HCAs, natural extracts from leaves, peels, and by-products of the agri-food industry are often used, as they are abundant in phenolic substances with antioxidant properties [50]. In fact, phenols can scavenge free radicals generated in several HCA synthesis processes [51]. Considering that OVW phenolic extracts are rich in 3,4-DHPEA-EA and 3,4-DHPEA [35], which have been shown to withstand grilling and other severe cooking conditions (such as deep- and pan-frying) [27,28,29,30,31,32,33,34,35,36,37,38,39,40,41,42,43,44,45,46,47,48,49,50,51,52,53,54,55], it was thus interesting to test OVW phenolic extracts for the prevention of HCAs’ formation.

Table 1 also reports the presence of 4 major HCAs (IQ, 8-MeIQx, 4,8-DiMeIQx, and PhIP). Only 8-MeIQx and 4,8-DiMeIQx were detected in C and P1 samples, with values ranging from 20.21–26.21 and 9.80–11.31 ng/patty, respectively. PE addition and storage length had no significant effect on the HCAs’ content. In fact, the HCAs levels found in the present study were very low, so the phenol-enriched samples and C did not show any statistically significant differences. This could probably be due to the “soft” cooking conditions (200 °C, 4 min per side), to which the patties were subjected. Indeed, in a study in which beef samples were previously marinated with different herbs and spices and then grilled at 240 °C/10 min [53], higher concentrations of polar and non-polar HCAs (207.2 ng/g meat) were detected in the control samples as compared to those found in our study. In the above study, PhIP, IQ, and 7,8-DiMeIQx were the most abundant polar HCAs, while MeIQ and MeIQx concentrations were below the detection limit in both the control and marinated samples [52]. Moreover, marination was able to inhibit the production of HCAs in grilled meat when individual herbs and spices and their combinations were used, except for PhIP in grilled beef marinated with curry leaf. According to Gibis and Weiss [51], in fact, phenolic compounds obtained from agri-food by-products act as radical scavengers, trapping free radicals generated in different pathways of HCA production during red meat grilling.

### 3.4. Mutagenicity and Genotoxicity of Stored-Cooked Beef Patties

Little is known about the mutagenicity and genotoxicity of grilled red meat in relation to the formation of COPs and HACs. In our study, we evaluated the cocktail effects of the two processing contaminants (COPs and HCAs) obtained from extracts of stored-grilled beef patties.

The genotoxicity of the extracts from stored-cooked beef patties formulated without added phenols (C) or enriched with OVW phenols (P1: 7.0 mg phenols/patty) were evaluated by the Comet assay on freshly isolated human PBMCs after exposure in complete RPMI medium at 37 °C for 30 min. The results presented in Table 2 show that the solvent (DMSO) did not induce DNA damage on the PBMC, whereas a clear effect was observed after treatment with the control meat extracts. In fact, the damage was approximately twice that of DMSO and was independent of storage time. The inclusion of phenols in the beef patties significantly reduced the DNA damage and this effect was more evident after 6 days of storage (Table 2). The genotoxicity-reducing effect in the P1 samples could be related to the indirect effect of phenols as inhibitors of the neo-formation of genotoxic compounds like COPs. On the other hand, the different meat extracts did not induce a significant increase of revertants in the Ames/Salmonella assay compared to the spontaneous revertants (Table 2). Although the inclusion of phenols slightly reduced the number of revertants, this effect was not statistically significant.

A range of in vitro and in vivo biochemical activities have been attributed to COPs, with relevant physiological and pathological implications [9]. The chemical structure of oxysterols makes them more polar and diffusible across cell membranes than cholesterol [8], thus making them more reactive. Indeed, several researchers have observed that COPs can promote atherogenicity, cytotoxicity, mutagenicity, carcinogenicity, inflammation, fibrosis, and programmed cell death in various cells and tissues [9]. Oxysterols may also contribute to the development of multiple sclerosis, age-related macular degeneration, and osteoporosis [9]. Therefore, considering all these biological effects, it is important to evaluate the efficacy and impact of natural antioxidants from vegetable extracts on the formation and activity of COPs. Biasi et al. [54] tested the anti-inflammatory properties of phenolic compounds found in Sardinian wine extracts, showing that these phytochemicals are able to protect CaCo-2 human enterocyte-like cells from oxysterol-induced cytokine production.

On the other hand, little information is available about the genotoxicity of HCAs on freshly isolated normal PBMC. While PhIP and IQ were tested for genotoxicity on human cryopreserved lymphocytes [55], the only data available on the genotoxic effect of HCAs on PBMC are those of Fuccelli et al. [56]. Their findings demonstrated that DNA damage was produced in PBMC by all the HCAs examined (PhIP, IQ, MeIQx, and DiMeIQx), and that this damage was exacerbated by the addition of a metabolic activator (S9-mix). In the case of DiMeIQx and MeIQx, the DNA repair inhibitors 1-fl-D-Arabinofuranosylcytosine (araC) and hydroxyurea (HU) also increased the genotoxicity. They also demonstrated that in the presence of phenolic compounds from extra virgin olive oil, olive leaf extracts, and olive extracts, the genotoxic effect of some HCAs (especially PhIP) was inhibited by phenolic extracts at low concentrations (0.1–1.0 µM). Shaughnessy et al. [56] instead analyzed the genotoxicity and mutagenicity of the extracts obtained from beef meat cooked at two different temperatures. The extract from meat cooked at 100 °C was not mutagenic, and no HCAs were detected therein. On the contrary, the extract from meat cooked at 250 °C showed a high content of HCAs, with PhIP as the most abundant compound; this extract was mutagenic, displaying higher potency in strain YG1024 compared to strain TA98.

### 3.5. Principal Component Analysis (PCA)

**Figure 1:**
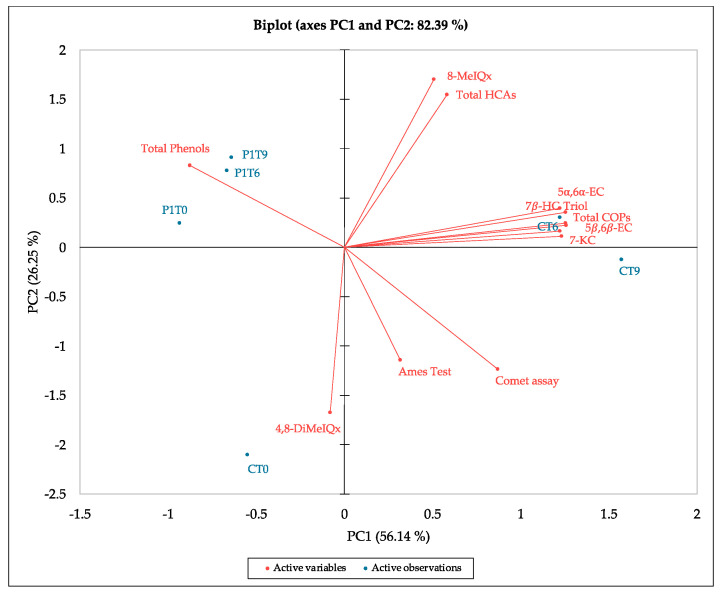
Biplot of all parameters. 4,8-DiMeIQx, 2-amino-3,4,8-trimethyl-3H-imidazo[4,5-f]quinoxaline; 5α,6α-EC, 5α,6α-epoxycholesterol; 5β,6β-EC, 5β,6β-epoxycholesterol; 7β-HC, 7β-hydroxycholesterol; 7-KC, 7-ketocholesterol; 8-MeIQx, 2-amino-3,8-dimethyl-imidazo[4,5-f]quinoxaline; HCAs, heterocyclic aromatic amines; Triol, cholestanetriol.

All data were subjected to PCA to identify the factors that were more determinant for evaluating the effects of PE addition and storage-cooking on beef patties (Figure 1). A total variance of 82.39% was explained by the first two components (56.14% for PC1 and 26.25% for PC2).

Total phenols are in the opposite quadrant (2) with respect to the mutagenicity (Ames test) and genotoxicity (Comet assay) parameters (quadrant 4), thus confirming an inverse correlation among them. The CT6 and CT9 samples are more correlated to all COPs and are in the same quadrant of total HCAs (except for 4,8-DiMeIQx), while P1 samples are in the same quadrant as total phenols. Regarding mutagenicity and genotoxicity parameters, they are located in the same quadrant of CT6 and CT9 samples, but they are not closely related.

The observed samples’ and parameters’ distribution in the biplot therefore explains how the phenolic extract can limit the formation of COPs, as well as reduce the risk of mutagenicity and cytotoxicity associated with the ingestion of grilled meat samples [18]. Moreover, while phenols and COPs are more correlated to PC1, HCAs are more separated on PC2. In fact, these compounds are known to cause DNA damage [9,57,58,59,60,61].

## 4. Conclusions

In conclusion, this preliminary study demonstrated that OVW phenols were able to reduce the accumulation of genotoxic compounds in stored-cooked beef patties. The added phenolic compounds progressively decreased due to both the storage and grilling to which the samples were subjected, but more than 20% of the added phenols were still present after 9 days in the display refrigerator. The retained phenols proved to be effective in reducing the formation of cholesterol oxides (4.59 times lower in P1 than in C samples) during the shelf-life of the patties. Regarding HCAs, very low levels (<40 ng/patty) were found in both C and P1 samples under the cooking conditions tested, indicating that the level of added PE did not affect the total HCA content. The Comet assay confirmed that patty extracts were genotoxic on PBMCs, and this effect was reduced by the inclusion of PE, while no mutagenicity was detected by the Ames test. In conclusion, this study demonstrated that PE obtained from the purification of OVW could be a promising approach for improving the oxidative stability and limiting the formation of potentially harmful compounds in stored-cooked beef patties, thus helping to reduce the cancer risk associated with the consumption of these products. To confirm these preliminary results, it would be necessary to test other PE addition levels to better define the suitable doses to counteract the formation/accumulation of these hazardous compounds. In addition, considering that few in vivo studies have been performed on meat products enriched with PE from OVW, it would be advisable to carry out more studies that are able to supply data about the ability of PE to contrast the effects of long-term exposure to oxysterols and HCA levels that could be physiologically important for humans.

## Figures and Tables

**Table 1:** Total phenols (mg/patty), single and total COPs (µg/patty), single and total HCAs (ng/patty) of cooked patties during storage. C (Control), meat batter + maltodextrins; P1, meat batter + 7.00 mg phenols/patty.

		Storage Time (Days)						SEM	*p*		
	Samples	0		6		9			F	S	F × S
Total phenols	C	-	B	-	B	-	B	0.52	***	***	***
	P1	1.62	a,A	0.92	b,A	0.48	c,A				
7β-HC	C	76.29	c,B	323.27	b,A	428.64	a,A	0.35	***	***	***
	P1	115.11	b,A	156.12	a,B	117.45	b,				
5β,6β-EC	C	51.55	c,B	172.47	b,A	283.72	a,A	0.34	***	***	***
	P1	67.75	b,A	84.59	a,B	67.66	c,B				
5α,6α-EC	C	17.00	c,B	101.29	a,A	77.98	b,A	0.22	**	N.S.	N.S.
	P1	18.96	b,A	27.26	a,B	30.84	a,B				
CT	C	8.73	c	24.75	a,A	21.38	b,A	0.12	**	N.S.	N.S.
	P1	8.35	b	10.64	a,B	9.07	b,B				
7-KC	C	76.15	c,B	324.02	b,A	547.61	a,A	0.63	***	***	***
	P1	109.03	b,A	116.14	a,B	80.78	c,B				
Total COPs	C	229.72	c,B	945.80	b,A	1359.33	a,A	0.82	***	***	***
	P1	319.20	b,A	394.74	a,B	295.80	c,B				
IQ	C	n.d.		n.d.		n.d.					
	P1	n.d.		n.d.		n.d.					
8-MeIQx	C	20.21	N.S.	26.21	N.S.	24.54	N.S.	0.01	N.S.	N.S.	N.S.
	P1	22.87	N.S.	24.86	N.S.	26.04	N.S.				
4,8-DiMeIQx	C	11.31	N.S.	9.80	N.S.	10.58	N.S.	0.00	N.S.	N.S.	N.S.
	P1	9.91	N.S.	10.06	N.S.	10.27	N.S.				
PhIP	C	n.d.		n.d.		n.d.					
	P1	n.d.		n.d.		n.d.					
Total HCAs	C	31.51	N.S.	36.01	N.S.	35.11	N.S.	0.01	N.S.	N.S.	N.S.
	P1	32.78	N.S.	34.92	N.S.	36.31	N.S.				

Results as reported as means and standard error means (SEM) of 2 or 3 independent replicates. a–c indicates significant differences (Tukey’s test; *p* ≤ 0.05) within the same sample during the shelf-life. A-B indicates significant differences (student’s *t*-test; *p* < 0.05) among different formulations. ** *p* < 0.01, *** *p* < 0.001. 4,8-DiMeIQx, 2-amino-3,4,8-trimethyl-3H-imidazo[4,5-f]quinoxaline; 5α,6α-EC, 5α,6α-epoxycholesterol; 5β,6β-EC, 5β,6β-epoxycholesterol; 7β-HC, 7β-hydroxycholesterol; 7-KC, 7-ketocholesterol; 8-MeIQx, 2-amino-3,8-dimethyl-imidazo[4,5-f]quinoxaline; CT, cholestanetriol; F, formulation; HCAs, heterocyclic aromatic amines; IQ, 2-amino-3-methyl-3H-imidazo[4,5-f]quinoline; n.d., not detected; N.S., non-significant; PhIP, 2-amino-1-methyl-6-phenylimidazo[4,5-b]pyridine; S, storage.

**Table 2:** Comet assay determined the genotoxic effect (expressed as % of DNA in the tail) on freshly isolated human PBMCs, while the Ames Test assessed the mutagenic effect (expressed as number of revertants per plate) on the S. typhimurium TA98 strain. Both effects were induced by diverse meat extracts (C, P1) after different storage time. C (Control), meat batter + maltodextrins; P1, meat batter + 7.00 mg phenols/patty.

		Storage Time (Days)						SEM	*p*		
	Samples	0		6		9			F	S	F × S
Comet Assay	DMSO	2.8	C	2.5	B	2.4	B	0.52	**	N.S.	N.S.
	C	5.8	A	5.7	A	6.1	A				
	P1	4.1	a,B	1.9	b,B	3.0	ab,B				
Ames Test	Spontaneous revertants	12.2	N.S.	12.2	N.S.	12.2	N.S.	1.32	N.S.	N.S.	N.S.
	C	20.5	N.S.	21.0	N.S.	13.5	N.S.				
	P1	13.5	N.S.	15.5	N.S.	11.5	N.S.				

Results as reported as means and standard error means (SEM) of 3 independent replicates. a-b indicate significant differences (student’s *t*-test; *p* ≤ 0.05) within the same sample during the shelf-life. A–C indicate significant differences (student’s *t*-test; *p* ≤ 0.05) among different formulations. ** *p*< 0.01. F, formulation; N.S., non-significant; S, storage.

## Data Availability

Data are contained within the article.
